# Association of Mask Mandates and COVID-19 Case Rates, Hospitalizations, and Deaths in Kansas

**DOI:** 10.1001/jamanetworkopen.2021.14514

**Published:** 2021-06-23

**Authors:** Donna K. Ginther, Carlos Zambrana

**Affiliations:** 1 Institute for Policy & Social Research, University of Kansas, Lawrence; 2 National Bureau of Economic Research, Cambridge, Massachusetts

## Abstract

This case-control study examines the association between counties that adopted state mask mandates in Kansas with COVID-19 cases, hospitalizations, and deaths.

## Introduction

This study examined the association between mask mandates in Kansas counties and COVID-19 cases, hospitalizations, and deaths. The Kansas executive order that took effect on July 3 was adopted by only 15 counties, and 68 counties did not have a mandate through October. A second mask mandate order took effect on November 25, and 40 additional counties adopted it.

## Methods

For this case-control study, data for the daily number of cases and deaths per county were from the *New York Times*^1^ and hospitalizations by county of residence were collected from the Kansas Department of Health and Environment^2^ (eMethods and eReferences in the Supplement). We adjusted the number of cases, hospitalizations, and deaths by each county’s 2019 population to obtain the rate per 100 000 and took a 7-day moving average of these variables. We refer to these population-adjusted rates as cases, hospitalizations, and deaths for the remainder of this report. This study was deemed not human subjects research by the University of Kansas institutional review board and followed the Strengthening the Reporting of Observational Studies in Epidemiology (STROBE) reporting guideline.

We used information from the Kansas Health Institute^3^ to classify counties by mask mandate and other restrictions (eTable in the Supplement). We limited our sample to 15 counties that always had a mask mandate (referred to as mask) as of July 10, 2020, and 68 counties that had no mandate (no mask) as of October 31, 2020. We estimated cases through December 4 because the governor’s November order caused mask mandate adoptions. Because hospitalizations and deaths lag COVID-19 cases, we estimated those through December 18.

We used linear regression difference-in-differences models.^4^ Cases were regressed on an indicator variable that starts 21 days after the mask mandate to allow for changes in mask-wearing behavior, an indicator for no COVID-19 cases, and the number of days since the first recorded case. Hospitalizations and deaths were regressed on lagged COVID-19 caseloads (hospitalizations 21 days and deaths 35 days). All models include controls for county and day fixed effects and use 95% CIs for statistical significance.

## Results

The Figure shows cases, hospitalizations, and deaths in mask and no mask counties in Kansas between March and December 18, 2020. At the time of the mask mandate, COVID-19 case rates in mask counties were 3 times higher than in no mask counties (15 cases per 100 000 population vs 5 cases per 100 000 population). These trends reversed, and by October 26 cases were 2.1 times higher in no mask counties (44 cases per 100 000 population vs 21 cases per 100 000 population). We see similar results for hospitalizations, with the rates in no mask counties being 1.4 times those in mask counties starting in mid-October (October 16: 2.6 hospitalizations per 100 000 population vs 1.8 hospitalizations per 100 000 population). Deaths were 1.8 times higher in no mask counties by November 1 (0.56 deaths per 100 000 population vs 0.32 deaths per 100 000 population).

**Figure.**
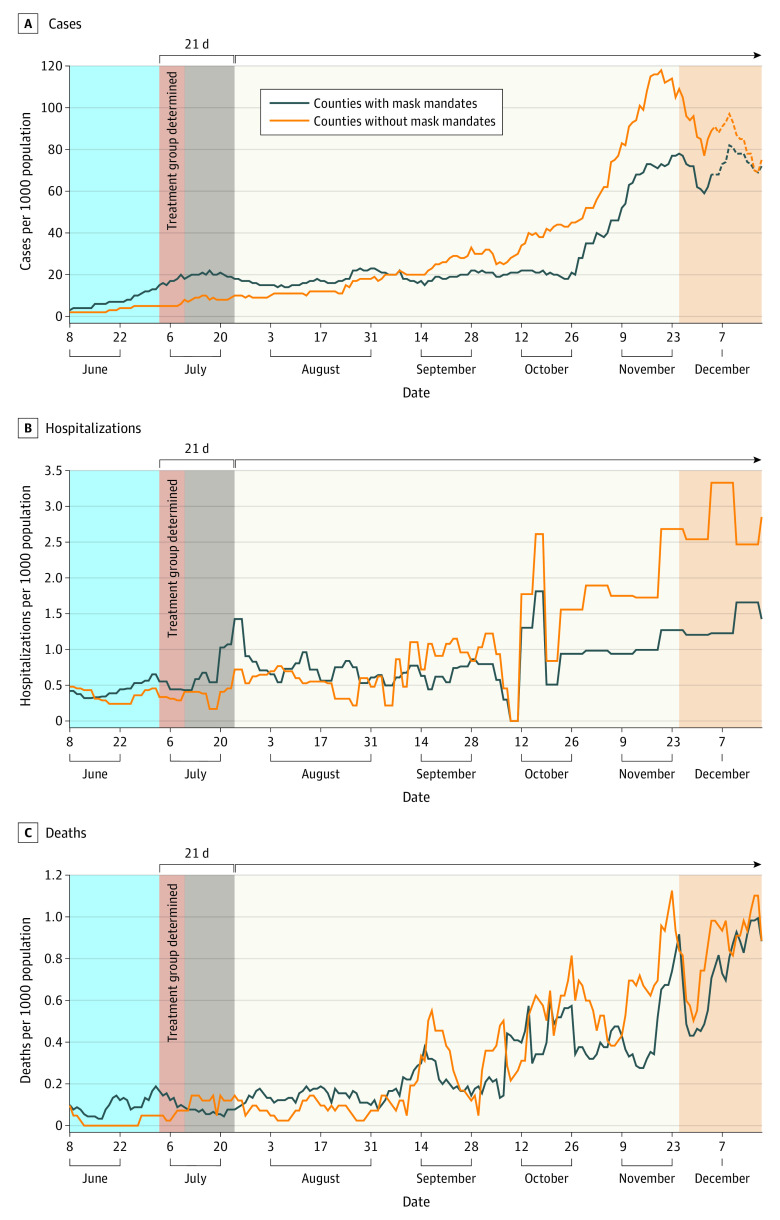
Seven-Day Moving Average of COVID-19 Cases, Hospitalizations, and Deaths per 100 000 Population in Mask and No Mask Counties in Kansas Dashed lines in panel A represent associations confounded by the November mask mandate.

The Table shows estimated associations between the counties with a mask mandate and number of COVID-19 cases, hospitalizations, and deaths. Cases were lower by 20.33 (95% CI, −26.54 to −14.12) per day in mask relative to no mask counties through December 4. This is equivalent to a 60% reduction in COVID-19 cases at the mean of 34.18 (95% CI, 33.31 to 35.06). Hospitalizations were lower by 0.81 (95% CI, −1.21 to −0.40) per day, a 60% reduction at the mean of 1.35 (95% CI, 1.30 to 1.39). Deaths were lower by 0.29 (95% CI, −0.51 to −0.08) per day, a 65% reduction from the mean of 0.45 (95% CI, 0.42 to 0.48). There were small differences in total cases between mask counties and for those with additional restrictions, such as limits on restaurants and gatherings.

**Table.** Linear Difference-in-Differences Analysis of Daily COVID-19 Cases, Hospitalizations, and Deaths per 100 000 Population in Counties With Mask Mandates Relative to No Mask Mandate

Variables	Estimated difference vs no mask counties/d (95% CI)^a^				
	Cases	Hospitalizations	Deaths	Mask only (cases)	Mask plus (cases)^b^
Mask mandate^c^	−20.33 (−26.54 to −14.12)	−0.81 (−1.21 to −0.40)	−0.29 (−0.51 to −0.08)	−21.27 (−27.24 to −15.30)	−19.72 (−27.22 to −12.23)
Days since first case	−0.31 (−0.44 to −0.18)	NA	NA	−0.32 (−0.45 to −0.19)	−0.31 (−0.44 to −0.18)
No cases	−8.11 (−20.64 to 4.43)	NA	NA	−7.66 (−19.73 to 4.40)	−8.17 (−20.81 to 4.48)
New cases					
21 d lag	NA	0.01 (0.00 to 0.01)	NA	NA	NA
35 d lag	NA	NA	−0.0008 (−0.004 to 0.002)	NA	NA
Constant	18.98 (10.67 to 27.28)	0.18 (−0.14 to 0.50)	0.09 (−0.07 to 0.25)	18.22 (9.89 to 26.55)	18.51 (10.11 to 26.92)
Mean	34.18 (33.31 to 35.06)	1.35 (1.30 to 1.39)	0.45 (0.42 to 0.48)	35.58 (34.58 to 36.57)	35.26 (34.31 to 36.20)
Case loads					
Observations, No.	14 940	16 102	16 102	12 960	13 680
Observed caseloads since July 24, No.	55 232	1782	707	26 212	25 382
Estimated caseload reduction	−35 230 (−45 995 to −24 465)	−1549 (−2322 to −775)	−562 (−967 to −157)	−18 015 (−23 072 to −12 959)	−15 447 (−21 316 to −9579)
Estimated caseload reduction (% of mean)	−59.5 (−77.6 to −41.3)	−60.1 (−90.2 to −30.1)	−65.1 (−112.0 to −18.2)	−59.8 (−76.6 to −43.0)	−55.9 (−77.2 to −34.7)

^a^ Case estimates are through December 4, when a new mask mandate was issued by the governor. Because of lag between COVID-19 cases and outcomes, hospitalizations and deaths are estimated through December 18.

^b^ Mask plus counties denotes counties that imposed additional restrictions, such as limits on sit-down restaurants and gatherings. Mask only and mask plus designations omit Crawford, Mitchell, and Montgomery counties.

^c^ We estimated the effect of the mask mandate starting 21 days after it was announced to allow for changes in mask-wearing behavior. Linear regression models include controls for day and county.

## Discussion

Counties that adopted the July mask mandate in Kansas experienced significantly lower rates of COVID-19 cases, hospitalizations, and deaths compared with those that did not. These findings corroborate previous studies that found that mask mandates slowed the growth of COVID-19 cases in Kansas counties^5^ and reduced the spread in states.^6^ Our results comparing mask-only policies with masks plus additional restrictions suggest that mask-wearing is associated with these reductions.

This study was limited because it did not control for daily testing rates by county in the state of Kansas, which were not available. Mask mandates are not the same as compliance, and our results should be considered lower-bound estimates of the association between mask-wearing and COVID-19. Our results suggest that mask mandates may provide an effective way to reduce cases of COVID-19, hospitalizations, and deaths.

## Notes

Supplement. eMethods. eReferences. **eTable.** Mask Mandates Adopted in Kansas. July 3-December 9, 2020
