# Supplementary material for: Association of Mask Mandates and COVID-19 Case Rates, Hospitalizations, and Deaths in Kansas
Source: JAMA Netw Open. 2021 Jun 23;4(6):e2114514. doi: 10.1001/jamanetworkopen.2021.14514 (PMC8223099; doi:10.1001/jamanetworkopen.2021.14514)
Supplement: Supplement. — eMethods. eReferences. eTable. Mask Mandates Adopted in Kansas. July 3-December 9, 2020 [file jamanetwopen-e2114514-s001.pdf]

Supplemental Online Content

Ginther DK, Zambrana C. Association of mask mandates and COVID-19 case rates, hospitalizations, and deaths in Kansas. *JAMA Netw Open.* 2021;4(6):e2114514. doi:10.1001/jamanetworkopen.2021.14514

eMethods.

eReferences.

eTable. Mask Mandates Adopted in Kansas. July 3-December 9, 2020.

This supplemental material has been provided by the authors to give readers additional information about their work.

© 2021 Ginther DK et al. *JAMA Network Open.*

eMethods.
Data:

Data for this study were obtained from various sources. We used the daily total number of cases per county from *The New York Times* COVID-19 Data in the United States GitHub repository.[1] We adjusted the number of cases by each county's 2019 population[2] to obtain the number of cases per 100,000 population. The daily data are noisy so we followed most reporting approaches by constructing a seven-day moving average of cases. Descriptive statistics are reported in eTable 1.

As Kansas embraced reopening in June 2020, COVID-19 cases increased, prompting the governor to issue Executive Order 20-52 establishing a state-wide mask mandate on July 3. We used a list of county official actions compiled by the Kansas Health Institute[3] to classify counties according to whether they adopted the state's mask mandate. The county policies are shown in eTable 2. Fifteen of the more populous counties followed the mask mandate (Mask). In eight counties, cities opted to follow the mask mandate but the surrounding county opted out (Partial Enforcement). Twenty-one counties adopted the mask mandate two or more weeks after the governor's first executive order (Late Adopters #20-52). A second mask mandate executive order took effect on November 25, and 40 additional counties adopted it (Late Adopters #20-68). We drop counties with Partial Enforcement and Late Adopters who imposed a mask mandate between July 11 and October 31, 2020. We drop Partial Enforcement counties because incomplete compliance with the mask mandate will bias the estimated effect of the mandate downwards. We drop Late Adopters to Executive Order #20-52 because we expect the late decision to adopt the mask mandate would be an endogenous choice in response to the number of cases in the county. Our estimation sample includes the 15 Mask counties that adopted the mask mandate (underlined) and the 68 No Mask counties in **bold** in eTable2 that adopted a mask mandate after October 31st or never had a mask mandate.

Statistical Analysis:

We estimated the association between the mask mandate and COVID-19 case rates using difference-in-differences (DID) models. This approach allows researchers to compare changes in case rates in the treatment (Mask) counties before and after the mandate to changes in case rates in control counties (No Mask) before and after mandate.[4] In our main model, we estimated a linear regression DID model where the dependent variables, the seven-day average of the daily number of COVID-19 cases, hospitalizations, and deaths per 100,000 population, were regressed on an indicator variable that starts 21 days after the mask mandate. We use this measure to allow time for the number of cases to reflect the change in mask-wearing behavior. Hospitalizations and deaths also included lagged COVID-19 caseloads (hospitalizations 21 days and deaths 35 days). The specifications include county fixed effects, day fixed effects, an indicator for no COVID-19 cases, and a variable for the number of days since the first recorded case in the county. This last variable can be considered county-specific time trends of COVID-19 cases. County fixed effects control for all time-invariant characteristics of the county that may be associated with case rates such as population, population density and economic conditions. Day fixed effects control for time-varying characteristics that are unobserved such as testing rates. All models use robust standard errors.

We estimate the intent to treat association of the mask mandate using this difference-in-differences study design. Therefore, we limited our analysis sample to counties that always had

© 2021 Ginther DK et al. *JAMA Network Open.*

a mask mandate (Mask) as of July 10, 2020 to counties that had not adopted a mask mandate (No Mask) as of October 31, 2020. Mask counties can be considered the treatment group and No Mask counties the control group.

We tested for the presence of pre-existing trends in our data. We repeated our main analysis with 14 days of pre-mask mandate lead variables. If these lead variables are statistically significant, this would indicate that there were pre-existing trends in the treatment and control counties that would confound our results. These estimates are reported in eTable 3. We reject the null hypothesis that the lead variables are jointly significantly different from zero for cases and deaths. There is no evidence of pre-existing trends for those outcomes. However, we reject the null hypothesis that the coefficients on hospitalizations are jointly significantly different from zero. Inspection of these estimates in eTable 3 indicates that nine of the 14 lead coefficients are positive. If we add these coefficients up their sum is 0.14, indicating that prior to the mask mandate, hospitalizations were increasing in mask mandate counties. Our estimates in Table 1 and eTable3 show that hospitalizations were lower after the mask mandate was adopted, reversing the pre-trend. This bolsters our argument that the mandate was associated with lower hospitalizations.

eReferences.

1. nyt-covid-19-bot. Coronavirus (Covid-19) Data in the United States. nytimes/covid-19-data. https://github.com/nytimes/covid-19-data. Accessed on October 12, 2020.
2. County Population Totals: 2010-2019. https://www2.census.gov/programs-surveys/popest/datasets/2010-2019/cities/totals/sub-est2019_20.csv.
3. https://www.khi.org/assets/uploads/news/15015/40830_table1sc.pdf
4. Wooldridge, JM. *Econometric Analysis of Cross Section and Panel Data*. Cambridge, MA: MIT Press; 2010.

eTable. Mask Mandates Adopted in Kansas. July 3-December 9, 2020.[a]

Mask	Partial Enforcement County Without (City with Mandate)[b]	Late Adopters (Order #20-52)	Late Adopters (Order #20-68)	Never Mask
Allen<br>Atchison[d]<br>Bourbon<br>Crawford<br>Dickinson[d]<br>Douglas[d]<br>Franklin[d]<br>Jewell<br>Johnson<br>Mitchell<br>Montgomery<br>Morris[d]<br>Saline[d]<br>Shawnee[d]<br>Wyandotte[d]	Cowley<br>Winfield(7/6)<br>Rice<br>Lyons(7/24)	Barber (10/21)[c]<br>**Brown (11/16)**<br>Clay (7/19)[c]<br>**Cloud (11/19)**<br>**Ellsworth (11/11)**<br>Geary (8/5)[c]<br>Gove (8/3)[c]<br>Greeley (10/13)[c]<br>Harvey (9/16)[c]<br>**Jefferson (11/11)**<br>Kearny (10/14)[c]<br>Lyon (11/6)[b]<br>Emporia (8/11)<br>**Marshall (11/16)**<br>McPherson (11/11)[b]<br>Lindsborg (7/20)<br>McPherson (10/26)<br>Moundridge (11/23)<br>**Nemaha (11/12)**<br>Pratt (7/16)[c]<br>Reno (7/15)[c]<br>Republic (8/4)[c]<br>Scott (8/10)[c]<br>Sedgwick (7/24)[b]<br>Wichita (7/3)<br>Stanton (7/23)[c]	**Barton (11/25)**<br>**Chase (11/25)**<br>**Cherokee (11/25)**<br>**Cheyenne (11/25)**<br>**Doniphan (11/25)**<br>Ellis (11/25)[b]<br>Hays (7/27)<br>**Finney (11/25)**<br>**Grant (11/25)**<br>**Gray (11/25)**<br>**Greenwood (12/8)**<br>**Hamilton (11/25)**<br>**Harper (11/25)**<br>**Jackson (11/30)**<br>**Kingman (11/25)**<br>**Kiowa (11/25)**<br>Labette (11/25)[b]<br>Parsons (7/6)<br>Marion (11/25)[b]<br>Marion (7/29)<br>Goessel (11/17)<br>Hillsboro (11/25)<br>Miami (11/25)[b]<br>Osawatomie (7/9)<br>Paola (7/14)<br>**Morton (11/25)**<br>**Norton (11/25)**<br>**Osborne (11/25)**<br>**Ottawa (12/7)**<br>**Pawnee (11/25)**<br>**Rawlins (11/25)**<br>Riley (11/23)[b]<br>Manhattan (7/7)<br>**Rooks (11/25)**<br>**Russell (11/20)**<br>**Sherman (11/25)**<br>**Smith (11/25)**<br>**Wabaunsee (11/25)**<br>**Washington (11/25)**<br>**Wilson (11/25)**	**Anderson**<br>**Butler**<br>**Chautauqua**<br>**Clark**<br>**Coffey**<br>**Comanche**<br>**Decatur**<br>**Edwards**<br>**Elk**<br>**Ford**<br>**Graham**<br>**Haskell**<br>**Hodgeman**<br>**Lane**<br>**Leavenworth**<br>**Lincoln**<br>**Linn**<br>**Logan**<br>**Meade**<br>**Neosho**<br>**Ness**<br>**Osage**<br>**Phillips**<br>**Pottawatomie**<br>**Rush**<br>**Seward**<br>**Sheridan**<br>**Stafford**<br>**Stevens**<br>**Sumner**<br>**Thomas**<br>**Trego**<br>**Wallace**<br>**Wichita**<br>**Woodson**
Population 1,293,299	Population 44,445	Population 684,903	Population 506,832	Population 383,835

[a] Data from the Kansas Health Institute. Underlined counties are in the treatment group and **Bold** counties are in the control group.
[b] Counties with partial enforcement. Cities in the county having a mandate listed below the county name with the date the city adopted the mandate. These are dropped from estimation sample.
[c] Counties in late adopters group. These are dropped from the estimation sample because mask adoption was likely in response to changes in case rates.
[d] Counties that are Mask-Plus that have policies in addition to mask mandates such as limitations on gatherings or restrictions on bars and restaurants.

© 2021 Ginther DK et al. *JAMA Network Open.*
